# Action of Nitrous Oxide

**Published:** 1873-11

**Authors:** 


*Action of Nitrous Oxide.*—The *Archives de Phys.* states that Joylet and Blanche have obtained the following results from their experiments on this subject. Chemically pure nitrous oxide will not support the respiration either of animals or plants, as they cannot decompose the gas. When breathed in a pure state by animals, it causes asphyxia and death, with all the symptoms usually occasioned either by strangulation or by the respiration of an inert gas, such as nitrogen or hydrogen. Nitrous oxide causes death in nearly the same time as these other asphyxiating agents. Nitrous oxide has no special anæsthetic action. The anæsthesia which it may produce when inhaled in a pure condition is only due to want of oxygen in the blood. Insensibility appears when the oxygen in arterial blood is reduced to less than 2 or 3 per cent. Arterial blood is then very dark, and contains 30 or 40 per cent. of nitrous oxide. Animals can live and show no alterations of sensibility while breathing mixtures of nitrous oxide and oxygen, in the same proportion as nitrogen and oxygen in air. The arterial blood then contains about 30 to 35 per cent. of nitrous oxide. Birds placed under a bell-jar filled with this mixture, behave exactly like those placed in a jar of the same size filled with air, and die after having exhausted the oxygen to a similar extent and formed a similar amount of carbonic acid. As nitrous oxide is an irrespirable gas, and does not possess the anæsthetic properties which have been attributed to it, the authors conclude that its employment cannot but be dangerous, and ought, on this account, to be excluded from medical practice.

